# Healing Right Way: A Stepped Wedge Cluster Randomised Controlled Trial Aiming to Enhance Quality of Life for Aboriginal Australian Survivors of Stroke and Traumatic Brain Injury

**DOI:** 10.1111/ajr.70106

**Published:** 2025-10-23

**Authors:** Elizabeth Armstrong, Tapan Rai, Judith M. Katzenellenbogen, Sandra J. Thompson, Meaghan McAllister, Natalie Ciccone, Deborah Hersh, Leon Flicker, Dominique A. Cadilhac, Erin Godecke, Graeme J. Hankey, Neil Drew, Colleen Hayward, Deborah Woods, Mel Robinson, Ivan Lin, Sanita Kratina, Jane White, Juli Coffin

**Affiliations:** ^1^ University Department of Rural Health South West Edith Cowan University Perth Western Australia Australia; ^2^ School of Mathematical and Physical Sciences University of Technology Sydney Sydney New South Wales Australia; ^3^ Cardiovascular Epidemiology Research Centre, School of Population and Global Health University of Western Australia Perth Western Australia Australia; ^4^ Western Australian Centre for Rural Health University of Western Australia Perth Western Australia Australia; ^5^ School of Medical and Health Sciences Edith Cowan University Perth Western Australia Australia; ^6^ Curtin School of Allied Health Curtin University Perth Western Australia Australia; ^7^ Medical School University of Western Australia Perth Western Australia Australia; ^8^ School of Clinical Sciences at Monash Health Monash University Melbourne Victoria Australia; ^9^ Sir Charles Gairdner and Osborne Park Healthcare Group Perth Western Australia Australia; ^10^ Centre for Neuromuscular and Neurological Disorders, Medical School University of Western Australia Perth Western Australia Australia; ^11^ Perron Institute for Neurological and Translational Science Perth Western Australia Australia; ^12^ Australian Indigenous HealthInfoNet, Kurongkurl Katitjin Edith Cowan University Perth Western Australia Australia; ^13^ Kurongkurl Katitjin Edith Cowan University Perth Western Australia Australia; ^14^ Geraldton Regional Aboriginal Medical Service Geraldton Western Australia Australia; ^15^ Ngangk Yira Institute for Change Murdoch University Murdoch Western Australia Australia

**Keywords:** aboriginal, clinical trial, indigenous, rehabilitation, stroke, traumatic brain injury

## Abstract

**Objective:**

To determine the effect of cultural security training (CST) for health professionals and access to an Aboriginal Brain Injury Coordinator (ABIC) for Aboriginal Australians with stroke or traumatic brain injury (TBI).

**Design:**

A stepped wedge cluster randomised controlled trial; the intervention package consisted of CST for hospital professionals and 6‐month access to ABICs providing education, support, liaison and advocacy; the commencement order of the intervention phase was randomised.

**Setting:**

Four urban and four rural hospitals in Western Australia, 2018–2022.

**Participants:**

Aboriginal adults ≥ 18 years hospitalised with stroke or TBI.

**Main Outcome Measures:**

Primary outcome was quality of life (Euro QOL–5D‐3L Visual Analogue Scale (EQ‐VAS)) score at 26 weeks post‐injury. Secondary outcomes were modified Rankin Scale, Functional Independence Measure, Hospital Anxiety and Depression Scale, Modified Caregiver Strain Index at 12 and 26 weeks, rehabilitation occasions of service, hospital compliance with minimum processes of care (MPC), acceptability of interventions, feasibility of ABIC role and costs.

**Results:**

In total, 108 participants recruited (target 312), 75% rural residents; 26‐week outcomes assessment completed for 78% of participants. The adjusted mean QoL showed no significant difference (*p* = 0.83). The MPC outcome favored the intervention group, adjusted difference in means 6.8% at 26 weeks, 95% CI (0.40%, 13.26%). There were no significant differences between control and intervention groups for other secondary outcomes.

**Conclusions:**

CST and implementation of an ABIC were feasible, acceptable and improved care processes for a predominantly rural population. Health outcomes did not differ. The effects of the COVID‐19 context are discussed.

**Trial Registration:**

ACTRN12618000139279

**Summary**

- What this paper adds
  ○ We report the first stepped wedge randomised controlled trial conducted in the area of brain injury in Aboriginal populations, with the first follow‐up data at 6 months for this predominantly rural‐located population.
  ○ The paper constitutes one of the few intervention studies conducted in the area of brain injury in Aboriginal populations.
  ○ This is the first clinical trial in the area and the first to evaluate the role of an Aboriginal Brain Injury Coordinator to assist with recovery.
  ○ The combination of cultural security training for health professionals and provision of an Aboriginal Brain injury Coordinator for Aboriginal Australians with stroke and traumatic brain injury within the Western Australian context was feasible, acceptable, safe and improved care provided across metropolitan, rural and remote areas.
  ○ This pragmatic real‐world trial of a complex intervention was able to be implemented and improved care despite significant challenges including the COVID‐19 epidemic.
  ○ The study provides a networked model for both future research and clinical services in metropolitan, rural and remote communities.
- What is already known on this subject?
  ○ There is a high incidence of stroke and traumatic brain injury in Aboriginal Australian communities.
  ○ Continuity of care post‐hospital discharge is sub‐optimal, with culturally secure care hampered by distance from Country and varying cultural awareness among hospital staff.
  ○ From the limited research conducted in this area to date, it has been recommended that more Aboriginal staff be involved in rehabilitation and that non‐Aboriginal clinicians receive cultural training.

## 1 Introduction

Aboriginal and Torres Strait Islander (respectfully Aboriginal) peoples in Australia who are hospitalised with stroke or traumatic brain injury (TBI) frequently experience isolation from their home, family and culture. Many live in regional, rural and remote areas [1, 2, 3] and are initially transferred to a metropolitan centre for acute care. Their care may be compromised by health service provider ignorance of Aboriginal culture, the impacts of colonisation and ineffective communication [4, 5, 6, 7].

This study was based in Western Australia (WA) where most Indigenous people are Aboriginal, yet culturally diverse, with multiple language groups living across the vast, sparsely populated state. We thus respectfully refer to Aboriginal (rather than Torres Strait Islander) peoples throughout. The varying cultural protocols, languages and traditions across the state are relevant to service provision planning and delivery.

The Healing Right Way (HRW) trial was developed in response to the recommendations of Aboriginal people with a stroke or TBI, [4, 5, 6] with high incidence of both in communities [1, 2, 3, 7, 8]. More culturally secure experiences during hospital stays following brain injury, including better communication between non‐Aboriginal health professionals and Aboriginal patients and increased support following discharge from hospital, were needed. Research showed more Aboriginal health professionals should be involved in rehabilitation care, and non‐Aboriginal clinicians requested further cultural training [9]. No previous clinical trials in rehabilitation and few intervention reports were noted for Aboriginal people after stroke or TBI.

The aim of the HRW multicentre trial was to develop, implement and evaluate an Intervention Package within WA to improve the quality of life for Aboriginal people with stroke or TBI within 26 weeks post‐injury compared to usual care. Our secondary hypotheses included significantly greater functional and emotional improvements in participants in the intervention group at 12 and 26 weeks and improvements in service delivery (outlined below). We also hypothesised that the package would be acceptable to Aboriginal participants and their families and health professionals and be feasible to implement.

The COVID‐19 pandemic began midway through the trial, affecting most of the intervention phase of the study. The trial continued throughout the pandemic. This paper includes reference to the effects of this extenuating circumstance on HRW and adaptations made to accommodate its effects.

## 2 Methods

The trial rationale and design have been previously described [10, 11]. Reporting is aligned with Consolidated Standards of Reporting Trials extension statement on the reporting of stepped wedge cluster randomised control trials (CRCTs) [12] and the Guidelines for Reporting Trial Protocols and Completed Trials Modified Due to the COVID‐19 Pandemic and Other Extenuating Circumstances: The 2021 CONSERVE Statement [13].

### 2.1 Study Design

Stepped wedge cluster randomised controlled trial. The trial design was based on principles aligned with an Aboriginal Research Framework [14] and Indigenous Standpoint Theory [15]. These included Aboriginal leadership; acknowledgment of colonisation as a social determinant of health/disability; acknowledgment of the diversity of Aboriginal communities; and Aboriginal community capacity building. See Appendix A for details of the trial's alignment with guidelines for health research involving Aboriginal and Torres Strait Islander peoples (the CONSIDER Statement Checklist) [16].

### 2.2 Setting

The eight hospital sites (four rural and four urban) were located in Western Australia.

### 2.3 Randomisation

Each rural site was paired to an urban site. Each pair was treated as a cluster, and each urban–rural cluster was randomised to one of four sequences which determined the order of commencement of the intervention phase (see study protocol for further detail) [10].

**FIGURE 1:**
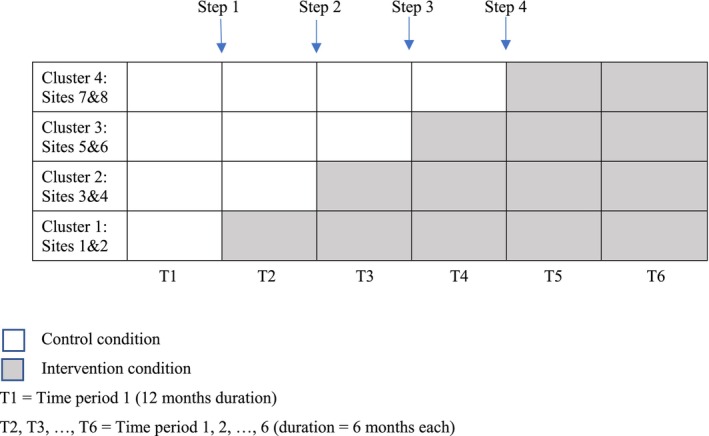
Stepped wedge design.

All sites commenced in the control phase, during which baseline data were collected (Figure 1). The first sequence crossed over into the intervention phase at the end of 52 weeks and remained in intervention for the remainder of the trial. The remaining sequences switched to the intervention phase at 26‐week intervals. Starting at 130 weeks, all sites were in the intervention phase, so that at the end of 156 weeks, each cluster had achieved at least 26 weeks of enrolment in the intervention phase.

During the trial, external factors impacted recruitment including the COVID‐19 pandemic and changes to next‐of‐kin consent legislation in WA [17]. Due to slower than predicted recruitment at the commencement of the trial, the initial six‐month step of the wedge was extended to 12 months.

### 2.4 Blinding

Participants and treating clinicians were unable to be blinded to the trial phase. All assessments were performed by clinically qualified, cultural‐security trained assessors blinded to the trial phase of the participant.

### 2.5 Participants

Aboriginal adults ≥ 18 years who presented within 6 weeks of an acute stroke or traumatic brain injury were eligible for the trial, provided they:

- Had a neurological deficit as determined by the National Institute of Health Stroke Scales (NIHSS) [18] (if they had a stroke) or
- Had a score of at least 8 on the Glasgow Coma Scale (GCS) [19] if diagnosed with TBI; and
- Were deemed able to benefit from rehabilitation by the medical or allied health team.

In the original protocol [10], the window of recruitment closed at 4 weeks post‐injury. This was increased to 6 weeks to enrol more patients who were initially very unwell but recovered sufficiently to be eligible for rehabilitation. All eligible patients from each site were invited to participate by clinical staff trained by the research team for this study. A hospital‐based Aboriginal Liaison Officer was involved where possible. Participants were enroled in the control or intervention group according to the study phase of their recruitment site. Enrolment of family members originally entailed wet‐ink signatures. Restrictions associated with COVID‐19 required an amendment to enable the option of verbal consent obtained over the telephone.

### 2.6 Baseline Characteristics

Baseline participant characteristics included recruitment region and demographics. Baseline clinical characteristics included injury type/severity, disability, anxiety, depression and functional independence [10].

### 2.7 Intervention

The intervention had two components. The first, cultural security training (CST), consisted of a three‐hour, face‐to‐face workshop and 3 h of online modules. These were developed by the research team members experienced in cultural training, with input from local Aboriginal Hospital Liaison teams in each of the regions where the training took place, and the Aboriginal Health Council of WA. The workshops were offered to health professionals (nursing, medical, allied health staff) every 6 months during the intervention period at each site; a minimum of 20 health professionals were trained at each site. Participants were envisaged as ‘champions’ and encouraged to model behaviours and attitudes to encourage other staff to follow the principles of the training. In addition, training was repeated so that new staff members had opportunities to attend over time.

The second component provided support of an Aboriginal Brain Injury Coordinator (ABIC) for participants in hospital and until 26 weeks post‐injury onset [20]. The ABIC role was developed by research team members in collaboration with participating Aboriginal Community Controlled Health Organisations and the Neurological Council of WA [20]. The role was designated for an Aboriginal health professional and included provision of education, support, liaison and advocacy services to participants and their families. The ABIC was required to be in face‐to‐face or telephone contact with the participant at least six times during the intervention period. ABICs were experienced health professionals and were local to the regions of participants, with training in working with people with ABI provided.

### 2.8 Follow‐Up

Participants were followed up prospectively and assessed at 12 and 26 weeks post‐injury either face to face or by telephone/telehealth as needed.

### 2.9 Outcomes

The primary outcome was quality of life, measured on the Euro QOL–5D‐3L VAS (EQ‐VAS) [21] at 26 weeks post‐injury. Secondary outcome measures were assessed at 12‐ and 26‐week post‐injury. Secondary participant‐level outcome measures included disability associated with brain injury as measured by the modified Rankin Scale—mRS [22] functional independence as measured on the Functional Independence Measure—FIM [23] and anxiety and depression, measured using the Hospital Anxiety and Depression Scale (HADS) [24] Participant satisfaction with their hospital experiences was also assessed via questionnaire at 12 and 26 weeks (see Appendix B). Caregiver strain was measured by the Modified Caregiver Strain Index [25] Outcome measures could be administered face‐to‐face or over the telephone. Serious adverse events (SAEs) were recorded throughout. System‐level outcomes at 12‐ and 26‐week post‐injury included percent of the minimum processes of care (MPC) achieved, number of allied health occasions of service and resource utilisation. MPC related to aspects of care received during inpatient admission, such as involvement of family, involvement of Aboriginal Liaison Officer, documentation of participant's first language, and use of interpreter if required [10] (see Appendix C for full list). Level of acceptability to hospital staff of the CST component of the intervention was obtained through custom‐designed questionnaires. As noted above, all assessments were undertaken by culturally trained assessors. We were unable to recruit Aboriginal assessors for these roles, reflecting the shortage of available Aboriginal health professionals.

### 2.10 Sample Size

The trial was designed to detect a between‐group difference of 15 points (standardised effect size of *d* = 0.6) on the EQ‐VAS. It was estimated that 312 participants (13 per cluster per time period) would be required to achieve 80% power at a significance level of 0.05. See the Statistical Analysis Plan for details [11].

### 2.11 Statistical Methods

Baseline characteristics were summarised using descriptive statistics. Age was summarised using median and inter‐quartile range, while other continuous variables were summarised using means and standard deviations; categorical variables were summarised using frequencies and percentages.

Outcome measures were treated as continuous, except for mRS, which was dichotomised as independent (0–2) or dependent/dead [3, 4, 5, 6]. Summary statistics were reported for the whole cohort and by trial phase (control/intervention).

The unadjusted outcomes were supplemented by results of linear mixed models, constituting the primary analysis. For the primary outcome measure (EQ‐VAS at 26 weeks), the model adjusted for age at recruitment, injury type, injury severity (dichotomised mRS at baseline), and time period as fixed effects, and recruitment site and participant as random effects. The models for the continuous secondary outcomes also adjusted for injury type, injury severity and time period as fixed effects, and recruitment site and participant as random effects. Since these outcomes were assessed at both 12‐ and 26‐week time points, the models also included time‐since‐injury as a fixed effect, together with the interaction of time‐since‐injury and trial phase (control/intervention). The interaction effect was used to estimate the adjusted 12‐ and 26‐week between‐group differences. A logistic generalised linear mixed model supplemented the results for the dichotomised mRS. Due to convergence issues, the confounders included in this model differed from those in the models for continuous outcomes, with time‐since‐trial‐commencement entered as a continuous variable.

The analyses of primary and secondary outcomes adjusted as above were conducted on an intention to treat (ITT) basis, where all participants were included in the phase (control/intervention) to which they were recruited, regardless of completion. These analyses are reported without imputation of missing data. ITT analyses with imputation of missing data were conducted separately, under the assumption that all missing data were missing at random (MAR). A sensitivity analysis was conducted to assess the MAR assumption (see Appendix D for details).

All analyses were conducted using the R Statistical Programming Language.

A concurrent process evaluation [26] was undertaken utilising the Consolidated Framework for Implementation Research [27] to explore contextual factors influencing specific trial processes, implementation of the intervention package and ultimate outcomes.

An economic evaluation to describe the potential cost‐effectiveness of the intervention is planned. This evaluation will be reported in a future publication.

### 2.12 Positionality Statement

Our multidisciplinary team included Aboriginal and non‐Aboriginal experts in the fields of brain injury, cardiovascular disease, cancer, dementia and health service planning. Four Aboriginal researchers were long‐term members of the research team, nine non‐Aboriginal researchers had lengthy experience collaborating on research with Aboriginal communities, and a further six were experienced in clinical trials. Further additional Aboriginal partners for example, Aboriginal Health Council of WA, Regional Aboriginal Health Consultants, hospital‐based Aboriginal liaison teams came on board to assist in the implementation to suit local contexts. The ABICs were central to the implementation, were interviewed for the process evaluation, co‐authored publications and led or participated in several state and national seminar, conference and media presentations.

### 2.13 Ethical Considerations

The Protocol for Healing Right Way was approved by Royal Perth Hospital (the central ethics committee for the study), St John of God Hospital, Edith Cowan University and the WA Aboriginal Health Research Ethics.

### 2.14 Role of Funding Source

The funders did not influence the study design, data collection, interpretation, or writing of the report in any way.

## 3 Results

### 3.1 Participant Flow

**FIGURE 2:**
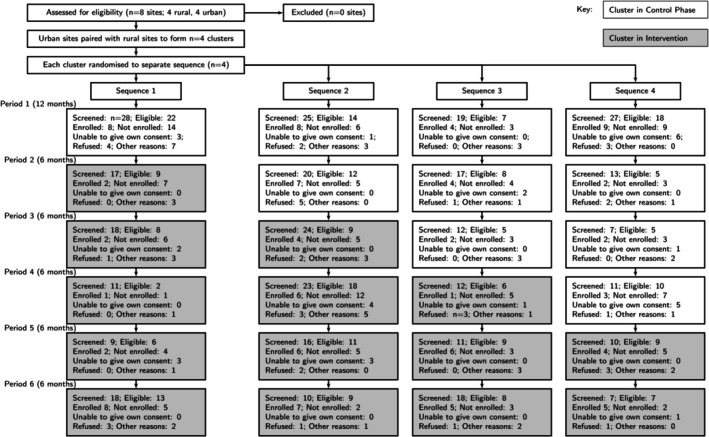
Flowchart of progress through phases healing right way stepped wedge trial.

During the trial period, 382 Aboriginal Australians who presented with acute stroke or TBI were screened from February 2018 to July 2021. Of these, 241 (63%) were eligible for the trial and 108 (45%) were enroled; 47 (43.5%) in the control phase and 61 (56.5%) in the intervention phase (Figure 2).

### 3.2 Recruitment

Recruitment was predominantly in the urban sites (*n* = 86; 79%), although 75% of participants resided in non‐metropolitan areas (Table 1). The median age of the cohort was 55 years. More males than females were recruited (59%), and stroke presentations predominated (*n* = 89; 82%). Half the participants were recruited before the onset of COVID‐19 and half after the onset. Reflecting the stepped‐wedge design, 84% of the intervention group (*n* = 51) were recruited after the onset of COVID‐19, as compared to 6% (*n* = 3) of the control group. While Aboriginal Liaison Officers in recruitment were aspired to, busy hospital Liaison teams and additional pressures due to COVID‐19 (including absences due to illness) made their involvement challenging, potentially impacting recruitment. Detailed discussion of recruitment is provided in the Process Evaluation paper [28].

**TABLE 1:** Participant demographics and baseline clinical characteristics.

	Control	Intervention	All
	*n* (%)	*n* (%)	*n* (%)
Recruitment region			
Metropolitan	35 (74.5%)	51 (83.6%)	86 (79.6%)
Regional	12 (25.5%)	10 (16.4%)	22 (20.4%)
Patient details			
Age median (IQR)	55.7 (15)	53.6 (20.2)	55.1 (14.9)
< 65	38 (81%)	50 (82%)	88 (81%)
65–80	8 (17%)	10 (16%)	18 (17%)
> 80	1 (2%)	1 (2%)	2 (2%)
Gender			
Male	26 (55%)	38 (62%)	64 (59%)
Female	21 (45%)	23 (38%)	44 (41%)
Other/not disclosed	0 (0%)	0 (0%)	0 (0%)
Place of residence prior to brain injury			
Perth metro	11 (23.4%)	16 (26.2%)	27 (25%)
Regional town	22 (46.8%)	35 (57.4%)	57 (53%)
Aboriginal community	11 (23.4%)	5 (8.2%)	16 (15%)
Not recorded	3 (6.4%)	5 (8.2%)	8 (7%)
Living arrangements prior to brain injury			
Home (alone)	5 (11%)	9 (14.8%)	14 (13%)
Home (with others)	41 (87%)	50 (82%)	91 (84%)
Supported accommodation, for example, nursing home, hostel	1 (2%)	0 (0%)	1 (1%)
No fixed home	0 (0%)	1 (1.6%)	1 (1%)
Other	0 (0%)	1 (1.6%)	1 (1%)
Injury type			
TBI	3 (6%)	16 (26%)	19 (18%)
Stroke	44 (94%)	45 (74%)	89 (82%)
Injury severity	N/A	mRS used	instead
Baseline mRS (binary)			
Independent (0–2)	7 (15%)	13 (21%)	20 (19%)
Dependent/dead (3–6)	40 (85%)	48 (79%)	88 (81%)
Baseline HADS			
Anxiety mean (SD)	7.8 (4.7) [*n* = 46]	7.7 (4.1) [*n* = 57]	7.8 (4.3) [*n* = 103]
Depression mean (SD)	5.6 (4.1) [*n* = 46]	6.1 (4.2) [*n* = 57]	5.9 (4.2) [*n* = 103]
Baseline FIM mean (SD)	91 (29)	92.7 (28.7)	91.9 (28.8)
Alcohol consumption (Yes)	20 (61%) [*n* = 33]	28 (74%) [*n* = 38]	48 (68%) [*n* = 71]
Drug use (Yes)	10 (30%) [*n* = 33]	11 (29%) [*n* = 38]	21 (30%) [*n* = 71]
Diabetes (Yes)	33 (70%)	34 (56%)	67 (62%)
Heart disease (Yes) [ischaemic heart disease]	23 (49%)	15 (25%)	38 (35%)

The baseline characteristics of the treatment groups (control/intervention) were balanced except for a larger proportion of participants with TBI in the intervention (*n* = 16; 26%) than in the control phase (*n* = 3; 6%). Participants in the intervention phase reported more symptoms of depression at baseline (Median HADS‐D = 6.0) than in the control phase (Median HADS‐D = 4.5). Diabetes and ischaemic heart disease were more common co‐morbidities in the control than in the intervention group. Table 2 provides primary language information that reflects the diversity of participants and language groups involved in the study.

**TABLE 2:** Languages spoken by participants.

Participant's primary language spoken	No.
English	73
Ngadju	1
Ngaatjatjara	1
Miriwoong	2
Ngarinyin	1
Walmajarri	6
Walpiri	1
Yulparija	1
Kija	1
Wajarri	2
Kukatja	1
Banyjima	1
Martuthunira	3
Ngarluma	1
Nyangumarta	2
Martuwangka	1
Nyungar	9
Not recorded	1

### 3.3 Follow‐Up

At the 12‐week assessment, 92 participants (85%) were available for clinical assessments—38 of control (81%) and 54 of intervention phase (88.5%) participants (see Table 3). At the 26‐week assessment, 34 (72%) control and 50 (82%) intervention participants were clinically assessed (see Table 4). Reasons for non‐availability included assessor challenges in contacting participants due to changing residences/contact numbers, illness, funerals, travel and reliance on busy relatives.

**TABLE 3:** Outcomes at 12 weeks.

	All mean (SD) *n* = 92^a^	Control mean (SD) *n* = 38^a^	Intervention mean (SD) *n* = 54^a^	Unadjusted analysis		Adjusted analysis	
				Mean difference (95% CI)	*p*	Mean difference (95% CI)	*p*
Primary outcome measure							
EuroQoL‐5D‐3L‐VAS	64.7 (18.8)	70.6 (19.3)	60.5 (17.4)	−10.1 (−17.9, −2.3)	0.02^b^	−2.2 (−20.1, 15.7)	0.81
Secondary participant‐level outcomes							
mRS (binary)	[*n* = 92]	[*n* = 39]	[*n* = 53]				
Independent (0–2) *n* (%)	36 (39%)	19 (49%)	17 (32%)	OR = 2.0	0.11	OR = 1.3	0.20
Dependent/dead (3–6) *n* (%)	56 (61%)	20 (51%)	36 (68%)	(0.8, 5.1)		(0.1, 14.9)	
FIM	105 (21) [*n* = 88]	106.2 (24.1) [*n* = 38]	104.0 (18.6) [*n* = 50]	−2.2 (−7.2, 11.7)	0.64	−6.5 (−23.6, 10.6)	0.45
HADS	[*n* = 79]	[*n* = 37]	[*n* = 42]				
HADS: anxiety	6 (3.8)	5.4 (3.6)	6.5 (4)	1.1 (−0.63, 2.8)	0.21	1.8 (−1.0, 4.6)	0.19
HADS: depression	4.7 (3.2)	4.1 (3.3)	5.3 (3.1)	1.2 (−0.2, 2.7)	0.09	2.9 (0.4, 5.5)	0.03^b^
Modified Caregiver Strain Index	8.8 (7.0) [*n* = 25]	6.3 (3.4) [*n* = 11]	10.7 (8.5) [*n* = 14]	4.4 (−0.8, 9.7)	0.09	—	—
System‐level outcomes	[*n* = 108]	[*n* = 47]	[*n* = 61]				
Proportion of minimum processes of care achieved	52% (18)	48% (20)	55% (16)	7% (−0.6, 14.1)	0.07	7% (0.1, 13.9)	52% (18)
Occasions of service	72.5 (76.6)	61.8 (61.7)	80.7 (85.9)	18.9 (−9.3, 47.0)	0.19	−0.84 (−33.3, 31.6)	0.96

^a^ Unless otherwise noted in specific rows, all statistics/analyses are based on these numbers.

^b^ Statistically Significant at the 5% significance level.

**TABLE 4:** Outcomes at 26 weeks.

	All mean (SD) *n* = 84^a^	Control mean (SD) *n* = 34^a^	Intervention mean (SD) *n* = 50^a^	Unadjusted analysis		Adjusted analysis	
				Mean difference (95% CI)	*p*	Mean difference (95% CI)	*p*
Primary outcome measure							
EuroQoL‐5D‐3L‐VAS	64.7 (19.6)	70.1 (21.0)	61.1 (17.9)	−11.0 (−17.8, −0.2)	0.046^b^	−1.7 (−17.6, 14.1)	0.83
Secondary participant‐level outcomes							
mRS (binary)	[*n* = 84]	[*n* = 38]	[*n* = 50]				
Independent (0–2) *n* (%)	34 (39%)	21 (55%)	13 (26%)	OR = 3.5 (1.3, 9.5)	0.008^b^	OR = 3.1	0.38
Dependent/dead (3–6) *n* (%)	54 (61%)	17 (46%)	37 (74%)			(0.3, 37.4)	
FIM	103.7 (23.6)	105.9 (26.9)	102.2 (21.3)	−3.7 (−14.7, 7.3)	0.51	−1.9 (−19.1, 15.2)	0.82
HADS	[*n* = 74]	[*n* = 33]	[*n* = 41]				
HADS: anxiety	6.4 (3.9)	6.0 (3.4)	6.7 (4.3)	0.7 (−1.0, 2.5)	0.41	1.7 (−1.1, 4.5)	0.24
HADS: depression	5.7 (4.2)	5.3 (4.3)	6.0 (4.0)	0.7 (−1.3, 2.6)	0.50	1.9 (−0.7, 4.6)	0.15
Modified Caregiver Strain Index	9.9 (8.0) [*n* = 21]	6.7 (6.9) [*n* = 10]	12.8 (8.1) [*n* = 11]	6.1 (−12.9, 0.7)	0.08^b^	—	—
System‐level outcomes	[*n* = 108]	[*n* = 47]	[*n* = 61]				
Proportion of minimum processes of care achieved	54% (18)	51% (19)	57% (16)	6% (−13.4, 1.0)	0.10	6.8% (0.4, 13.3)	0.038^b^
Occasions of service	81.4 (108)	69.7 (69.7)	90.4 (102.6)	20.7 (−12.2, 53.7)	0.21	4.23 (−26.2, 34.6)	0.78

^a^ Unless otherwise noted in specific rows, all statistics/analyses are based on these numbers.

^b^ Statistically Significant at the 5% significance level.

### 3.4 Outcomes

In the unadjusted analysis, the control group achieved a higher quality of life at 26 weeks post‐injury (mean EQoL‐VAS 70; SD = 21) than the intervention group (mean EQoL‐VAS = 61; SD = 18). This unadjusted difference was statistically significant (*p* = 0.046) and similar to EQoL‐VAS scores recorded 12 weeks post‐injury. However, after controlling for age at recruitment, injury type/severity and recruitment site (the primary analysis), the adjusted mean EQoL‐VAS was 62.5 for the control group and 60.8 for the intervention group. The mean difference of 1.7 was not statistically significant (*p* = 0.83).

Similarly, a lower proportion of control participants displayed injury‐related disability (51% dependent/dead) than the intervention group (68% dead/dependent) at 12 weeks (unadjusted). This trend increased to a statistically significant difference at 26 weeks (Control: 45% dependent/dead; Intervention: 74% dependent/dead; *p* = 0.005). These differences were mitigated by our pre‐specified adjusted analyses, which showed that the differences were not statistically significant at either time point.

Both groups showed improvement in functional independence at 12 and 26 weeks compared to baseline. The between‐group differences at these timepoints were not statistically significant at either time point. The adjusted analysis indicated that the control group reported significantly fewer symptoms of depression at 12 weeks than the intervention group. However, at 26 weeks, there was no significant difference between groups.

### 3.5 Service Level Outcomes

A higher proportion of MPCs was achieved in the intervention (unadjusted mean = 55%; SD = 16 at 12 weeks) than in the control group (unadjusted mean = 48%; SD = 20); the unadjusted difference in means was not statistically significant (*p* = 0.07). The unadjusted mean difference was sustained but still statistically non‐significant at 26 weeks (intervention: mean = 57%, SD = 17; control: mean = 51%; SD = 22; *p* = 0.09). After adjusting for age, injury type and severity, time period and site, there was a statistically significant between‐group difference of 7% in the percent of MPCs achieved (*p* = 0.048) at 12 weeks. The difference was similar at 26 weeks (6.8%, *p* = 0.038). Between‐group differences on occasions of service at 12 weeks or 26 weeks post‐injury were not significant.

Of participant respondents to the hospital experience survey (*n* = 81 at 12 weeks and *n* = 66 at 26 weeks; 54 at both time points), the vast majority (86%) from both intervention and control groups had positive experiences at both time points, although two participants in each group reported very bad experiences. Both groups reported similar satisfaction with their hospital experience at week 12, but intervention group experiences were significantly more positive than control group experiences by week 26 (*p* = 0.04).

There was a relatively large amount of missing data predominantly related to the Modified Caregiver Strain Index, the HADS and the economic evaluation information. A lack of family member recruitment, often due to the dislocation of the person from family initially, contributed to the missing carer data. Some economic details were too difficult for participants to recall, were considered intrusive, for example, regarding financial matters, or were omitted due to participant fatigue.

### 3.6 Fidelity

The Intervention Package was implemented broadly as planned. All 61 intervention group participants received the services of an ABIC, although only 59% received ≥ 6 ABIC contacts (Range = 1–19). The CST reached the target attendance numbers for the face‐to‐face component (250 staff trained), although only 50% of attendees completed the online modules thereafter. This coincided with pressures resulting from COVID‐19, technological issues, staff workload and the completion not being compulsory. Aboriginal facilitators were involved in all training, with additional Aboriginal hospital staff contributions in the workshops.

### 3.7 Feasibility and Acceptability

Implementation of the ABIC role was acceptable and feasible. Feasibility was inferred through the successful employment, training and retention of Aboriginal staff to each of the ABIC positions across the state. During times of change/absence of staff, the team worked as a network to ensure that participants in all regions obtained the ABIC services either face‐to‐face or by phone. The survey of the hospital experience, to which the assessor was blinded as to intervention status and therefore could not directly address the ABIC intervention, was one measure through which acceptability was inferred. Other measures included agreement to have ABIC follow‐up visits (all participants agreeing to these visits). In regard to the CST, detailed surveys with participants in the training were conducted and revealed overwhelmingly positive responses; feasibility was also determined by attendance at face‐to‐face workshops and participation of Aboriginal cultural experts (predominantly local to the region) as co‐facilitators at all sessions. Interviews with project staff and facilitators also provided critical analysis of feasibility and appropriateness of the CST approach.

Besides providing cultural training for 250 health staff, HRW contributed significant research/clinical capacity building for Aboriginal and non‐Aboriginal staff across WA. Nine Aboriginal staff were recruited and trained as ABICs; three Aboriginal health professionals as research assistants; 46 non‐Aboriginal staff as assessors and 21 as Research Site Contacts (primary contact at the hospital recruiting site).

### 3.8 Adverse Events

During the trial, 51 serious adverse events were recorded. The proportion of participants who experienced at least one serious adverse event was significantly higher in the control group (*n* = 17; 36%) than in the intervention group (*n* = 9; 15%) (*χ* ^2^ = 6.66; *p* < 0.01). See Appendix E for further detail.

## 4 Discussion

Healing Right Way was the first brain injury related RCT within Aboriginal populations and the first to document 26‐week follow‐up of Aboriginal patients, including functional outcomes and quality of life. It was conducted in a complex, real‐world health service context, spanning metropolitan and rural areas, multiple health service providers and study partners, and a culturally diverse population. The RCT showed no significant quality of life or clinical differences between control and intervention groups, although MPC were significantly more adhered to in the intervention phases and participant‐reported hospital experiences were more positive in the intervention group. These results are suggestive only, as target numbers required to power the study to generate definitive results were not reached. Nevertheless, the findings provide important documentation and analysis of processes and experiences of care for Aboriginal people after brain injury, which is rarely recorded or focused upon to date.

The COVID‐19 pandemic starting midway into the recruitment period significantly affected all aspects of the study. As alluded to above, the involvement of Aboriginal Liaison Officers in the recruitment process was challenging, potentially affecting recruitment. Besides affecting recruitment, the pandemic was in progress for most of the intervention period, potentially negatively affecting the quality of life and clinical outcome measures in intervention compared with control periods. The infrastructural, logistical, community and health system impacts of the pandemic may have adversely affected the ability to receive family support and the ability to access health and rehabilitation services during this period [29]. The possibility that not everyone may want or need the kind of assistance offered by the ABICs must be considered in terms of the results; further qualitative feedback from participants using yarning methodology for assessing outcomes (as discussed in the Limitations section below) could have enhanced our understanding of this component of the intervention.

The fact that most participants were recruited in the metropolitan area but resided in regional, rural, or remote areas is consistent with previous studies [1, 2, 3] and reflects the need to focus on rehabilitation services in rural areas and to better address the transition of patients back to these areas after acute treatment in the metropolitan area. While the relatively young age of participants who had suffered a stroke and the high incidence of comorbidities were commensurate with other studies, it highlights the relatively complex recovery journeys that many Aboriginal people must navigate. These issues need consideration if relevant, accessible and flexible rehabilitation services are to be offered.

The follow‐up rate of participants was relatively high, given distances involved and oft‐cited anecdotal clinical reports of difficulty in follow‐up. Integral to the follow‐up in HRW was the use of existing and the establishment of new networks with Aboriginal health service providers and within Aboriginal communities across the state, that assisted the research team in locating and connecting with participants. Networks included hospital Aboriginal Liaison Officers, community services, Aboriginal Medical Services and other family members, where available. Strong relationships facilitated all study processes.

The finding of higher compliance with MPC in the intervention group suggests that CST and the presence of ABICs in hospitals can make a difference to the delivery of care. While simply running the trial may have heightened hospital staff's attention to care delivery to Aboriginal patients, the important role of training was supported by the positive response of staff to the cultural training provided and recommendations for more regular training.

The importance of a concurrent process evaluation cannot be underestimated, with ongoing feedback on trial processes informing practical modifications, while also providing implications for service delivery and future research methods. Translation and knowledge exchange occurred throughout the study, with ongoing meetings with, and presentations to clinical partners, policy advocates, community and Aboriginal people with brain injury and regular media engagement. Details are provided elsewhere [30].

In terms of generalisability of study processes and results, the presence of the pandemic during the study presented a unique set of circumstances. Nevertheless, findings related to the geographical and cultural context of the trial and many of the challenges encountered are relevant to and generalisable in the planning of future rehabilitation services. The systems used within the study to address the challenges faced are highly relevant to the provision of services across geographically large distances and in a context where patients are often flown from rural and remote areas to metropolitan areas for acute care. Findings regarding the importance of the ABIC in a ‘navigator’ role, methods for conducting rehabilitation assessments online rather than face to face, and networking methods used in HRW for follow‐up purposes should be highly generalisable.

### 4.1 Limitations

Diminished recruitment and sample size reduced the power of the study and the ability to detect an intervention effect, with no definite conclusions possible. The secondary outcomes are not adjusted for multiple comparisons, and any statistically significant results should be interpreted with caution. Low recruitment of TBI participants reflects challenges in reaching this group in the acute stage and limits conclusions about them. Besides COVID, the WA legislative changes surrounding consent had a significant impact on recruitment. Limited involvement of Aboriginal people in recruitment (logistical issues), poor systems for identification of Aboriginal patients in hospitals, and competing commitments of many hospital staff all influenced recruitment (all explored in the process evaluation companion paper) [28].

The stepped wedge design has inherent limitations in that changes occurring over time external to the trial itself may differentially influence outcomes between control and intervention periods. Although not all participants received the minimum number of ABIC visits, flexibility to varying needs and personalised care was needed. Multiple assessment tools, often inducing fatigue and reflecting the need for standardised, widely used assessments in RCTs, some tools were not culturally appropriate. Both contributed to missing outcome data and that the assessors collecting the outcome data were non‐Aboriginal could also have influenced results. Further qualitative feedback on the ABIC service (beyond the limited general survey) to assess participants' specific satisfaction with and perceptions of benefit from the intervention would have been useful. Future intervention studies could utilise a yarning framework in addition to culturally appropriate tools to capture quantitative outcome measures. The COVID‐19 context influenced availability of staff to attend the face‐to‐face cultural training and complete the online modules, both non‐compulsory. While the cultural training was positively received and the target numbers were reached, the numbers may have been too low for training to have affected participants' journeys and outcomes, particularly in large metropolitan hospitals where numbers and staff turnover are high.

## 5 Conclusion

The study was unable to definitively address the effect of this intervention on quality of life. The difference between control and intervention periods in compliance with MPC suggests that system‐level change is possible, with individual‐level change perhaps more challenging to determine. Despite challenges, HRW demonstrated that the intervention package was feasible and acceptable, and that a complex trial across multiple metropolitan and rural sites is possible in an Aboriginal health context. It has highlighted the importance of Aboriginal leadership, partnerships across sites, and community networks in following patients post‐discharge to enable the provision of comprehensive and culturally secure services.

## Author Contributions

**Elizabeth Armstrong:** conceptualization; formal analysis; funding acquisition; investigation; methodology; supervision; writing – original draft preparation; writing – review and editing. **Tapan Rai:** conceptualization; formal analysis; funding acquisition; methodology; writing – original draft preparation; writing – review and editing. **Judith Katzenellenbogen:** conceptualization; funding acquisition; methodology; writing – review and editing. **Sandra Thompson:** conceptualization; funding acquisition; methodology; writing – review and editing. **Meaghan McAllister:** project administration; methodology; resources; writing – review and editing. **Natalie Ciccone:** conceptualization; funding acquisition; methodology; writing – review and editing. **Deborah Hersh:** conceptualization; funding acquisition; methodology; writing – review and editing. **Leon Flicker:** conceptualization; funding acquisition; methodology; writing – review and editing. **Dominique Cadilhac:** conceptualization; funding acquisition; methodology; writing – review and editing. **Erin Godecke:** conceptualization; funding acquisition; methodology; writing – review and editing. **Graeme Hankey:** conceptualization; funding acquisition; methodology; writing – review and editing. **Neil Drew:** conceptualization; funding acquisition; methodology; writing – review and editing. **Colleen Hayward:** conceptualization; funding acquisition; methodology; writing – review and editing. **Deborah Woods:** conceptualization; funding acquisition; methodology; writing – review and editing. **Mel Robinson:** methodology; writing – review and editing. **Ivan Lin:** conceptualization; funding acquisition; methodology; writing – review and editing. **Sanita Kratina:** data curation; methodology; resources; software; writing – review and editing. **Jane White:** data curation; methodology; software; writing – review and editing. **Juli Coffin:** conceptualization; funding acquisition; methodology; writing – review and editing.

## Disclosure

The authors have nothing to report.

## Conflicts of Interest

The authors declare no conflicts of interest.

## Figures and Tables

**FIGURE C1:**
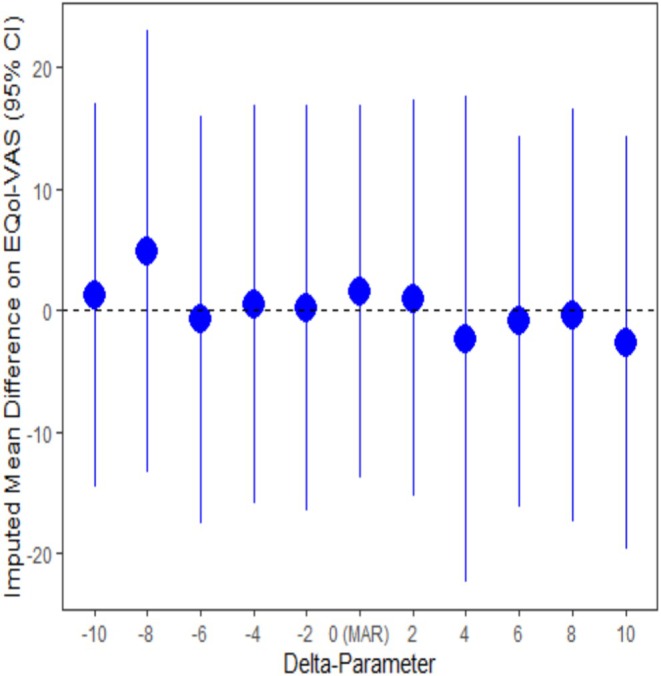
Effect of delta‐parameter adjustment on difference in EQ‐VAS at 26 weeks between intervention and usual care.

## Data Availability

The data from the study will not be shared due to the sensitivity surrounding the population involved in the study, including issues of data sovereignty. The ethical approval also does not include sharing individual‐level data.

## Appendix A Healing Right Way Addressing the CONSIDER Statement

**Table:**

GOVERNANCE 1. Describe partnership agreements between the research institution and Indigenous‐governing organisation for the research, (e.g., Informal agreements through to MOU (Memorandum of Understanding) or MOA (Memorandum of Agreement)). Five Aboriginal Community Controlled Health Organisations (ACCHOs) were part of the NHMRC Partnership Grant funding for Healing Right Way and committed in‐kind support to the project including professional support for the Aboriginal Brain Injury Coordinators employed on the project, office workspace and computer services at the ACCHO, and ongoing regular liaison with the research team throughout the study. The Aboriginal Health Council of WA was also a partner in the study, providing Aboriginal facilitators for the Cultural Security Training component of the study intervention. ACCHOs involved were: Geraldton Regional Aboriginal Medical Service; Kimberley Aboriginal Medical Services, Broome; Broome Regional Aboriginal Medical Service; Bega Garnbirringu Health Services, Kalgoorlie; Warrika Maya Health Service, Port Hedland. 2. Describe accountability and review mechanisms within the partnership agreement that addresses harm minimization. Regular meetings were held with all Aboriginal partner organisations either face to face or online (during COVID) to review the project, in particular the role of the Aboriginal Brain Injury Coordinator and how this role was working within their organisation including satisfaction with the amount of support being provided to the Coordinators from the research team. 3. Specify how the research partnership agreement includes protection of Indigenous intellectual property and knowledge arising from the research, including financial and intellectual benefits generated (e.g., development of traditional medicines for commercial purposes or supporting the Indigenous community to develop commercialization proposals generated from the research). The Aboriginal Investigators in Healing Right Way were central to research agreements and ensured that all plans regarding intellectual property ensuing from the project for example, journal publications, conference presentations were aligned with an Aboriginal Research Framework. This ensured Aboriginal authorship, co‐authorship, presentation and co‐presentations as deemed appropriate. Study results have been fed back to all partners as well as related senior Aboriginal policy makers and service planners in order to ensure that benefits to Aboriginal people with brain injury and their families/communities receive maximum benefit (see below). No financial benefits were involved in the study.
PRIORITISATION 4. Explain how the research aims emerged from priorities identified by either Indigenous stakeholders, governing bodies, funders, non‐government organisation(s), stakeholders, consumers and empirical evidence Healing Right Way was directly based on previous research with Aboriginal brain injury survivors and their families conducted by the research team over a 12 year period. This included an AIATSIS‐funded pilot study (Armstrong et al., 2012, 2015) and the Missing Voices project (Armstrong et al., 2019a,b). Both these projects also involved close partnerships with ACCHOs across Western Australia who informed researchers of local cultural and clinical needs of communities across the state. The key messages from these studies included the need for (i) Aboriginal health professional support both during acute hospitalisation and after hospital discharge and (ii) increased cultural security within current acute hospital and rehabilitation services, requiring more cultural training for hospital staff. The research team has also led the investigation of brain injury rehabilitation for Aboriginal people providing some of the first epidemiological data on traumatic brain injury on which to base Healing Right Way, along with national incidence figures published in recent years.
RELATIONSHIPS 5. Specify measures that adhere and honour Indigenous ethical guidelines, processes, and approvals for all relevant Indigenous stakeholders, recognising that multiple Indigenous partners may be involved, for example, Indigenous ethics committee approval, regional/national ethics approval processes. Ethics approval obtained from WA Aboriginal Health Ethics Committee Meetings with, and letters of support for ethics approval obtained from all Aboriginal organisation partners (ACCHOS) and Aboriginal Regional Health Planning Fora (Midwest Aboriginal Health Planning Forum, Pilbara Aboriginal Health Planning Forum, Kimberley Aboriginal Health Planning Forum) Aboriginal leadership of the research (3 key Aboriginal researchers on the research team) Ongoing regular meetings with all Aboriginal organisations involved Ongoing regular meetings with senior Aboriginal health service planners and managers (CEOs and senior staff of ACCHOs and Regional Aboriginal Health Consultants) Aboriginal Hospital Liaison teams closely involved with the project at all hospital research sites, assisting with recruitment and ensuring cultural safety of processes Aboriginal health professionals employed as Aboriginal Brain Injury Coordinators to maximise the cultural security for Aboriginal participants Interpreter services acquired for Aboriginal participants as required recognising prime importance of language needs in culturally secure health service delivery Employment of an Aboriginal cultural mentor for the Aboriginal Brain Injury Coordinators later in the trial as the need for this became more obvious 6. Report how Indigenous stakeholders were involved in the research processes (i.e., research design, funding, implementation, analysis, dissemination/recruitment). Four Aboriginal researchers were long‐term members of the research team. Additional Aboriginal research input was incrementally gathered as the project was designed. The research investigator team contributed to the original design, while additional Aboriginal partners for example, Aboriginal Health Council of WA came on board to assist in the design of the intervention that is, the cultural security workshop content, the workings of the Aboriginal Brain Injury Coordinator role. Regional Aboriginal Health Consultants were involved in the implementation phases, facilitating both Aboriginal staff and participant recruitment in all regions as well as assisting in the organisation of cultural workshops and sourcing of local Aboriginal facilitators. Aboriginal hospital liaison teams became increasingly important in the project as it progressed, providing local contextual information for the research team, participating in the cultural workshops and facilitating participant recruitment and follow‐up. The Aboriginal Brain Injury Coordinators were central to the project, providing ongoing feedback to the investigator team regarding local contexts and needs of participants. They provided interviews for the process evaluation, but were also involved in authoring publications and have led and/or participated in several state and national seminar, conference and media presentations. 7. Describe the expertise of the research team in Indigenous health and research. Our multidisciplinary team included Aboriginal and non‐Aboriginal experts in the fields of brain injury, cardiovascular disease, cancer, dementia and health service planning. Four Aboriginal researchers were long‐term members of the research team, nine non‐Aboriginal researchers had lengthy experience collaborating on research with Aboriginal communities, and a further six were experienced in clinical trials. Further additional Aboriginal partners for example, Aboriginal Health Council of WA, Regional Aboriginal Health Consultants, hospital‐based Aboriginal liaison teams came on board to assist in the implementation of the to suit local contexts. The Aboriginal Brain Injury Coordinators were central to the implementation as well as providing interviews for the process evaluation and co‐authoring publications and leading or participating in several state and national seminar, conference and media presentations.
METHODOLOGIES 8. Describe the methodological approach of the research including a rationale of methods used and implication for Indigenous stakeholders, for example, privacy and confidentiality (individual and collective) Described in main text. 9. Describe how the research methodology incorporated consideration of the physical, social, economic and cultural environment of the participants and prospective participants. (e.g., impacts of colonisation, racism and social justice). As well as Indigenous worldviews. Described in main text and in forthcoming Process Evaluation paper.
PARTICIPATION 10. Specify how individual and collective consent was sought to conduct future analysis on collected samples and data (e.g., additional secondary analyses; third‐parties accessing samples (genetic, tissue, blood) for further analyses). Ethics approval was only sought for analysis of the Healing Right Way data for purposes related directly to this project. The data is not being shared on any public databases and will not be used for further analyses. 11. Describe how the resource demands (current and future) placed on Indigenous participants and communities involved in the research were identified and agreed upon including any resourcing for participation, knowledge and expertise Extensive discussions at the planning stage within the research team involving the Aboriginal Investigators, as well as with Aboriginal organisations who were potential partners on the project were undertaken. 12. Specify how biological tissue and other samples including data were stored, explaining the processes of removal from traditional lands, if done and of disposal. Not applicable
CAPACITY 13. Explain how the research supported the development and maintenance of Indigenous research capacity (e.g., specific funding of Indigenous researchers). All Aboriginal research staff outside the Chief Investigator team, were remunerated by being employed on the project for example, Aboriginal Brain Injury Coordinators, cultural workshop facilitators, interviewers. All employed staff were trained in specific research methods. Nine Aboriginal staff (enroled nurses, Aboriginal health workers), received training to fill the Aboriginal Brain Injury Coordinator role, involving education around brain injury and research methods involved in randomised controlled trials and data recording using the REDCap database. Ongoing clinical and academic support was provided throughout the trial. Interviewers were trained in research methods surrounding RCTs and interviewing skills as needed. Authorship on publications/conference presentations is considered for all substantial contributions, with all of these having Aboriginal representation. The Coordinators in particular have been key authors and presenters at state and national conferences/seminars/media outlets. 14. Discuss how the research team undertook professional development opportunities to develop the capacity to partner with Indigenous stakeholders? The Aboriginal and non‐Aboriginal research team is a long‐standing team, with members working together for over 10 years. Both the Aboriginal and non‐Aboriginal members of the team have worked with Aboriginal stakeholders (community members, service providers and policy‐makers) for a number of years across a variety of research projects and with a variety of Aboriginal research colleagues. The researchers take every opportunity to attend relevant conferences and seminars surrounding the latest recommendations and issues involved in undertaken research with Aboriginal communities, and engage in ongoing active dialogue with Aboriginal research partners and community organisations.
ANALYSIS & INTERPRETATION 15. Specify how the research analysis and reporting supported critical inquiry and a strength‐based approach that was inclusive of Indigenous values. All of the research team members are committed to a strengths‐based approach to analysis and reporting. However analysis and interpretation of all data is overseen by the Aboriginal Investigators on the research team to ensure that this approach is accurately reflected in all publications and presentations.
DISSEMINATION 16. Describe the dissemination of the research findings to relevant Indigenous governing bodies and peoples. Feedback sessions with the Aboriginal Brain Injury Coordinators regarding study results were held across January 2022 – online and face to face. Feedback sessions to the Executive Committees of the participating WA Country Health Service sites outlining key findings of the study were provided later in 2022/early 2023. The Regional Aboriginal Health Consultants in these WACHS regions were part of the Executive Committee meetings. Feedback and translation sessions were held with WA Health Strategic Aboriginal Health Group in 2023, with the Director of Aboriginal Health Policy Directorate, WA Health, in attendance. Topics of discussion included results of the study, key findings, opportunities to translate the Aboriginal Brain Injury Coordinator role and potential business cases. There were 13 invited presentations to specific Aboriginal audiences and/or teams with Aboriginal representation throughout the study including conference presentations and media works. 17. Discuss the process for knowledge translation and implementation to support Indigenous advancement (e.g., research capacity, policy, investment). Healing Right Way's Knowledge Translation Plan involves three translational and knowledge exchange domains, relating to the role of Aboriginal Brain Injury Coordinators, cultural training of hospital staff and the research process itself. Knowledge plans were developed for key audiences, with potential translation products to be monitored for ongoing impact. Translation and knowledge exchange were iteratively embedded throughout the project life cycle via community engagement, partnership meetings and interviews. Activities included presentations to diverse audiences including bureaucrats, community and participants as noted above. Knowledge translation and implementation activities are ongoing through meetings with senior bureaucrats in the WA Department of Health and ACCHOs—in particular to recommend the sustained implementation of the Aboriginal Brain Injury Coordinator roles within health services. Discussion with national brain injury research teams around successful and culturally secure Healing Right Way research processes is continuing to stimulate interest, with national projects increasingly seeking advice on optimal methodologies for ensuring strengths‐based and culturally secure projects. Aboriginal colleagues are increasingly being invited to be part of brain injury research discussions and teams. The research team continues to build Indigenous research capacity through ongoing involvement of Aboriginal research staff on Healing Right Way in study presentations and publications, and continuing to employ these and new Aboriginal staff members in new projects related to brain injury.

## Appendix B Participant Questionnaire

**Table:**

Tell us what you think about…
Being in hospital and then coming home

Tick the box you think is right for you…

**Table:**

	Yes—it was good	In the middle	No—it was not good
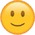
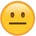
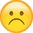
Looking back… how I was looked after in hospital			
How staff yarned with me and answered my questions			
How staff talked with my family			
How staff planned for what I wanted when I left hospital			
Receiving the information I needed			
Getting therapy if I needed it			
Preparing me for keeping busy and seeing friends			

Anything else you want to add?

Thank you for telling us how it has been for you.

## Appendix C Minimum Processes of Care (MPC)

- Patient assessed by allied health within 48 h of admission.
- Aboriginal Liaison Officer involved during inpatient stay.
- Language(s) spoken noted in medical file.
- Interpreter used if Aboriginal language is participant's primary language.
- Telephone or face to face contact with family made by any allied health staff during inpatient stay.
- Aboriginal brain injury educational resource provided during inpatient stay.
- Inpatient allied health service provided (any discipline).
- Discharge plan developed with patient and family at family conference.
- Outpatient allied health service provided (any discipline).
- In the case of transfer from metropolitan to rural service, verbal contact made by the metropolitan hospital with local rural service provider (hospital and local Aboriginal Medical Service).
- In the case of transfer from metropolitan to rural service, discharge report from metropolitan hospital sent to rural service provider (hospital and local Aboriginal Medical Service).

## Appendix D Missing Data Imputation and Sensitivity Analysis

### Introduction

As specified in the Statistical Analysis Plan (SAP), the primary analysis was conducted without imputing any missing data. However, data on most outcomes were available only for 92 of the 108 enroled participants (85%) at 12 weeks and 84 participants (78%) at 26 weeks. This appendix describes the process undertaken to examine the effect of the missing data and to assess the robustness of the result.

### Imputation of Missing Data

Missing data was initially imputed using chained equations, under the assumption that data was missing at random (MAR). Specifically, we assumed that the primary outcome measure (EQ‐VAS at 26 weeks) could reasonably be estimated from other observed variables. The variables we used in this imputation were:

- Age at the time of stroke
- Gender
- Hospital Site
- Intervention Phase (control/intervention)
- Time Period (time between commencement of trial and recruitment—in 6‐month blocks)
- Recruitment Step (of the stepped wedge design)
- Brain Injury Type (Stroke/TBI)
- mRS at baseline
- FIM at baseline
- HADS(D) at baseline
- HADS(A) as baseline
- Total number of Occasions of Service to 12 weeks post‐injury
- Applicable MPCs achieved to 12 weeks post‐injury
- mRS at Week 12 post‐injury
- FIM at Week 12 post‐injury
- HADS(D) at Week 12 post‐injury
- HADS(A) as Week 12 post‐injury
- EuroQoL‐VAS as Week 12 post‐injury
- mRS at Week 12 post‐injury
- FIM at Week 12 post‐injury
- HADS(D) at Week 12 post‐injury
- HADS(A) as Week 12 post‐injury

Five imputed datasets were generated with these predictors. The pre‐specified linear mixed model, which controlled for injury type, mRS at baseline, mRS at Week 26, time period, and age as fixed effects, and site as a random effect, was used to assess quality of life at 26 weeks, as measured by the EuroQoL‐VAS on each imputed dataset. The results of these analyses were pooled.

The pooled results estimated the difference between the usual care cohort and the intervention cohort (on EuroQoL‐VAS at 26 weeks) to be 1.5 (favouring intervention). Although the direction of this difference differed from that of the primary analysis, it was not statistically significant (*p* = 0.18).

### Sensitivity Analysis

The robustness of the ‘missing at random’ assumption was then tested using a delta‐parameter. The delta‐parameter is a measure of deviation from the ‘missing at random’ assumption. In this procedure, values of EQ‐VAS at 26 weeks that were imputed under the ‘missing at random’ assumption were adjusted by values of delta ranging from −10 to 10. Five imputed data sets were generated for each value of delta, and the results of the pre‐specified linear mixed model on these sets were pooled. Results of these pooled analyses for different values of delta are presented in an imputation plot.

### Imputation Plot

Figure C1 shows the effect of a delta‐parameter adjustment on the difference between the intervention and usual care cohorts on EQ‐VAS at 26 weeks. The central value on the x‐axis (delta = 0) represents imputation under the assumption of ‘missing at random’. The y‐axis represents the predicted difference on EQ‐VAS between the high intensity and low intensity cohorts at 26 weeks (the primary hypothesis). As seen from the graph, the predicted difference remains stable even with large values of delta (large deviations from the assumption of ‘missing at random’), and the difference remains small and statistically non‐significant. This shows that the estimated outcome is robust against fairly large variations of the ‘missing at random’ assumption.

## Appendix E Serious Adverse Events

**Table:**

	Control (*n* = 47)	Intervention (*n* = 61)	All (*n* = 108)
Total number of SAEs	31	20	51
Participants with 0 SAEs	30	52	82
Participants with 1 SAE	10	5	15
Participants with 2 SAEs	2	2	4
Participants with more than 2 SAEs	5	2	7

*Note:* All recorded Adverse Events were considered ‘Serious Adverse Events’ (SAEs); 48 of the SAEs (29 Control; 19 Intervention) were determined to be unrelated to the trial; the remaining 3 SAEs (2 Control; 1 Intervention) were considered ‘Unlikely to be related’ to the trial.
